# Deletion of the transcription factor Prox-1 specifically in the renal distal convoluted tubule causes hypomagnesemia via reduced expression of TRPM6 and NCC

**DOI:** 10.1007/s00424-020-02491-1

**Published:** 2020-11-16

**Authors:** Christina Schnoz, Sandra Moser, Denise V. Kratschmar, Alex Odermatt, Dominique Loffing-Cueni, Johannes Loffing

**Affiliations:** 1 Institute of Anatomy, University of Zurich, Winterthurerstrasse 190, CH-8057 Zurich, Switzerland; 2 Division of Molecular and Systems Toxicology, Department of Pharmaceutical Sciences, University of Basel, Basel, Switzerland; 3 National Centre of Competence in Research “Kidney.CH”, University of Zurich, 8057 Zurich, Switzerland

**Keywords:** Kidney, Renal distal convoluted tubule (DCT), DCT adaptation, Transcription factor Prox-1, NaCl cotransporter (NCC), Transient receptor potential cation channel subfamily M member 6 (TRPM6)

## Abstract

The renal distal convoluted tubule (DCT) is critical for the fine-tuning of urinary ion excretion and the control of blood pressure. Ion transport along the DCT is tightly controlled by posttranscriptional mechanisms including a complex interplay of kinases, phosphatases, and ubiquitin ligases. Previous work identified the transcription factor Prox-1 as a gene significantly enriched in the DCT of adult mice. To test if Prox-1 contributes to the transcriptional regulation of DCT function and structure, we developed a novel mouse model (NCC^cre^:Prox-1^flox/flox^) for an inducible deletion of Prox-1 specifically in the DCT. The deletion of Prox-1 had no obvious impact on DCT structure and growth independent whether the deletion was achieved in newborn or adult mice. Furthermore, DCT-specific Prox-1 deficiency did not alter DCT-proliferation in response to loop diuretic treatment. Likewise, the DCT-specific deletion of Prox-1 did not cause other gross phenotypic abnormalities. Body weight, urinary volume, Na^+^ and K^+^ excretion as well as plasma Na^+^, K^+^, and aldosterone levels were similar in Prox-1_DCT_^KO^ and Prox-1_DCT_^Ctrl^ mice. However, Prox-1_DCT_^KO^ mice exhibited a significant hypomagnesemia with a profound downregulation of the DCT-specific apical Mg^2+^ channel TRPM6 and the NaCl cotransporter (NCC) at both mRNA and protein levels. The expression of other proteins involved in distal tubule Mg^2+^ and Na^+^ handling was not affected. Thus, Prox-1 is a DCT-enriched transcription factor that does not control DCT growth but contributes to the molecular control of DCT-dependent Mg^2+^ homeostasis in the adult kidney.

**Supplementary Information:**

The online version contains supplementary material available at 10.1007/s00424-020-02491-1.

## Introduction

The renal distal convoluted tubule (DCT) is crucial for the control of ion homeostasis and the regulation of arterial blood pressure [28]. Although only 5–10% of the sodium (Na^+^) filtered at the glomerulus is reabsorbed in the DCT, the Na^+^ transport activity of the DCT is the highest of all nephron portions, which is reflected by a very high mitochondrial density in DCT cells. The Na^+^ transport in the DCT is driven by the basolateral Na^+^-K^+^-ATPase and critically depends on the activity of the thiazide-sensitive NaCl cotransporter (NCC) in the apical plasma membrane of DCT cells [28, 30]. The DCT is also important for the control of renal magnesium (Mg^2+^) excretion as the DCT is the sole site for active transcellular Mg^2+^ reabsorption along the nephron. The Mg^2+^ reabsorption in the DCT is mediated by the apical transient receptor potential cation channel subfamily M member 6 (TRPM6) [46] and modulated by the γ-subunit of the Na^+^-K^+^-ATPase encoded by the FXYD2 gene [2, 27].

Genetic diseases affecting the DCT evidence the significance of the DCT for the control of ion homeostasis and blood pressure [28]. In Gitelman syndrome, loss-of-function mutations in NCC lead to an autosomal recessive renal tubulopathy characterized by renal salt-wasting with low blood pressure, hypokalemic alkalosis, hypocalciuria, and hypomagnesemia [40]. Conversely, an inappropriate increase in NCC activity due to mutations in NCC-regulating with no lysine (*K*) kinases (WNK1 and 4) or ubiquitin ligase complexes (KLHL3, CUL3) causes salt-sensitive familial hyperkalemic hypertension (FHHt) with metabolic acidosis and hypercalciuria, also known as Gordon syndrome or pseudohypoaldosteronism type II (PHA II) [12, 28, 41]. Likewise, mutations in TRPM6 [36, 48] and FXYD2 [29] cause renal tubulopathies with magnesium wasting and hypomagnesemia [45].

The last couple of years brought tremendous insights into the molecular signaling mechanism that control the function of the DCT. A complex network of various kinases, phosphatases, and ubiquitin ligases was shown to regulate the DCT function. For example, the STE20-related proline-alanine–rich kinase (SPAK) and the oxidative-stress-responsive 1 kinase (OSR1) are known to directly phosphorylate and thereby activate NCC [13]. The activity of SPAK and OSR1 itself is controlled by the upstream kinases WNK1 and WNK4 [51] that again are regulated via the ubiquitin ligase complex proteins kelch-like 3 (KLHL3) and cullin 3 (CUL3) [13, 41]. The abundance of NCC is also directly regulated by ubiquitinylation via Nedd4–2 and the upstream kinase Sgk1 [34]. Phosphatases such as protein phosphatase 1 (PP1), 3 (PP3 or calcineurin), and 4 (PP4) can counteract the activity of the kinases and inactivate NCC [11]. Similar to NCC, the activity of TRPM6 is also controlled by several kinases. For example, the stimulatory effect of the epidermal growth factor (EGF) on TRPM6 is thought to be mediated by both Src-Family Kinases and mitogen-activated kinases (MAPK) and their downstream mediator PI3-kinase [43]. Likewise, AKT/PKB and Cdk5 were implicated in the insulin-dependent regulation of TRPM6 [45].

Despite this significant progress, we still lack a comprehensive understanding of the regulatory mechanisms that govern the DCT. In particular, we lack significant information about the transcriptional control mechanism. Genetic studies linked the hepatocyte nuclear factor 1 beta (HNF1b) and its cofactor PCBD1 to the regulation of TRPM6 [1, 20], but the role of other DCT-expressed transcription factors is less clear. Previously, we identified the transcription factor Prospero Homeobox 1 (Prox-1) as a gene that is about 100 times more abundant in the DCT than in in the sum of all other renal tubules of adult mouse kidney [33]. Together with NCC and TRPM6, Prox-1 belongs to the 20 gene products most enriched in the mouse DCT suggesting a specific role in this tubule segment. Also in the adult rat kidney, Prox-1 appears to be predominately if not exclusively expressed in the DCT as indicated by a comprehensive database on RNAseq data from isolated renal tubules [23].

Prox-1 is a transcription factor containing an evolutionary conserved homeobox-prospero domain that binds DNA and RNA and allows transcriptional regulation of other genes. It plays an essential role during developmental processes such as cell proliferation, cell fate determination, and progenitor cell differentiation in numerous mammalian organs (lymphatic system, CNS, eye, heart, liver, and pancreas) [8, 10, 32, 49]. Moreover, alterations in Prox-1 expression have been associated with several human malignancies including hematological diseases; brain tumors; and carcinomas of colon, pancreas, and liver, and the biliary system [10, 32]. In the kidney, Prox-1 is highly expressed in distal tubules of the developing mouse kidney where it is involved in differentiation and outgrowth of the medullary part of the loop of Henle [17]. As in the adult kidney, Prox-1 is enriched in the DCT [23, 33], we hypothesized that Prox-1 may contribute to the transcriptional control of DCT function. As a constitutive Prox-1 knockout is embryonically lethal [50], we developed a novel mouse model that allowed an inducible, DCT-specific deletion of Prox-1 in the adult kidney.

## Material and methods

### Animals

**Fig. 1:**
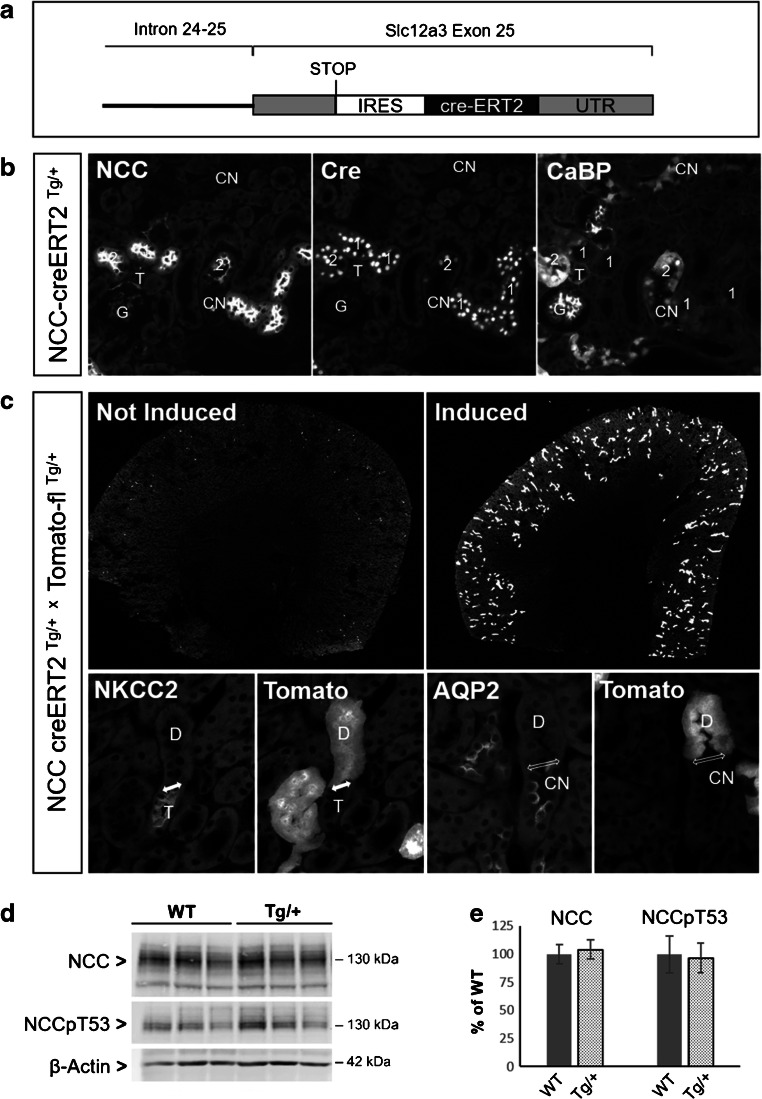
Characterization of NCC-cre-ERT2 mice. **A** Schematic representation of insertion of the tamoxifen-inducible creERT2 in the untranslated region (UTR) of the last exon of the Slc12a3/NCC gene. **B** Immunostaining for NCC, cre, and calbindin D28K (CB28) on consecutive cryosections from NCC-cre-ERT2 mice. Tamoxifen induces strong nuclear localization of the cre recombinase in NCC-positive DCT1 (1) and in DCT2 (2) with strong NCC and CB28-immunostaining. **C** Overviews on kidneys from NCC-cre-ERT2/tomato-flox mice (upper panel) show a strong tomato-related fluorescence in a kidney of tamoxifen-induced but not in the kidney of an uninduced mouse. High-power magnification (lower panel) demonstrate that the tomato-related fluorescence is strictly limited to the DCT (D) and is not found in the preceding NKCC2-positive TAL (T) and the downstream localized AQP2-positive CNT (CN). The segment transitions are indicated by double-headed arrows. Two pairs of consecutive cryosections. **D** and **E** Immunoblots and densitometry reveal that the creERT2 transgene does not interfere with NCC abundance and phosphorylation. Data are mean ± SEM, *n* = 3

Mice with “floxed” alleles for Prox-1 were a kind gift from Dr. G. Oliver (Chicago, USA). In this conditional knockout mouse model, exons 2 and 3 of the Prox-1 gene are flanked by loxP sites. Cre recombinase mediated deletion of these two exons removes the homeodomain and Prox domain and renders Prox-1 transcriptionally inactive [14]. To target specifically the DCT, a mouse model with a tamoxifen-inducible cre activity in the DCT was custom-made by the company Ozgene (Bentley, Australia). In these mice, the cre-ERT2 was introduced into the three prime untranslated region (3′-UTR) of the Slc12a3/NCC gene, where it is expressed using the same internal ribosomal entry site (IRES) as the Slc12a3/NCC gene (Fig. 1A). TdTomato-red reporter mice [26] were obtained from The Jackson Laboratories Repository. All experiments involving living animals were conducted in accordance with Swiss laws and approved by the veterinary administration of the Canton of Zurich (Kantonales Veterinäramt), Switzerland.

### Tamoxifen administration

Tamoxifen induction was performed in male mice 1–2 weeks after weaning (i.e., at the age of 35–45 days). Tamoxifen (Sigma-Aldrich, T5648) was dissolved in EtOH: sunflower oil (1:10). A dose of 2 mg per day was administered to the mice via gastric gavage on 5 subsequent days, 6 weeks prior to the experiments. The same dosage and procedure were applied to breastfeeding mother mice to induce cre expression in the newborn offspring.

### Plasma and urine ion measurement

#### Blood collection

Blood was sampled by puncture of the inferior vena cava of isoflurane-anesthetized animals. Whole blood was centrifuged for 10 min at 3000 *g* and plasma was removed subsequently.

#### Plasma Na^+^, K^+^, and Ca^2+^ measurement

Blood ions (Na^+^, K^+^, Ca^2+^) were measured with the ABL80Flex Blood Gas Analyzer (Radiometer, Copenhagen, Denmark).

#### Plasma and urine Mg^2+^ and PO_4_^3−^ measurement

Plasma and urine Mg^2+^ and PO_4_^3−^ measurements were performed by the Zurich Integrative Rodent Physiology (University of Zurich) using photometric methods (UniCel DxC 800, Beckman Coulter, Brea, CA, USA).

#### Urine Na^+^, K^+^, and Ca^2+^ measurement

Urine was collected for 24 h from mice kept individually in metabolic cages (Tecniplast S.p.A., Buguggiate, Italy). Urinary electrolytes (Na^+^, K^+^, Ca^2+^) were measured by flame photometry (EFOX 5053, Eppendorf, Hamburg, Germany). Urine creatinine was assessed by the Jaffe method.

### Aldosterone, 11-deoxycorticosterone, and corticosterone measurement

Aldosterone, 11-deoxycorticosterone, and corticosterone were determined by ultra-performance liquid chromatography-MS/MS (UPLC-MS/MS) as described previously with minor adaptations [42] using an Agilent 1290 Infinity II UPLC coupled to an Agilent 6495 triple quadrupole mass spectrometer. Data acquisition and quantitative analysis was performed by MassHunter (Version B.10.0. Build 10.0.27, Agilent Technologies).

### Kidney harvesting and tissue processing

#### Immunofluorescence

The kidneys of isoflurane-anesthetized adult mice were fixed by retrograde abdominal aortic perfusion of 3% paraformaldehyde (PFA) followed by rinsing with 0.1 M phosphate buffer (pH 7.4, 300 mOsm) and paraffin embedding. The kidneys of 10-day-old isoflurane-anesthetized pups were immersion fixed with paraformaldehyde (PFA) 3% for 12–16 h, then incubated in 0.1 M phosphate buffer (pH 7.4, 300 mOsm) for 4 h and finally embedded in paraffin.

#### Western blot and quantitative reverse transcription PCR

For western blot analysis and RNA extraction, perfusion was performed with PBS (pH 7.4) via the left ventricle of the heart; kidneys were removed and frozen in liquid nitrogen.

### Immunohistochemistry

Tissue blocks were sectioned at 4 μm on a standard rotary microtome (Microm HM355S, Thermo Fisher Scientific, Waltham, MA, USA). The Pathisto AS-2 automatic slide stainer (Pathisto GmbH, Garbsen, Germany) was utilized for deparaffination using Histo-clear and for tissue rehydration using serial ethanol washes. For epitope retrieval, slides were heated in 10 mM citrate buffer (pH 6.0) in a microwave (98 °C, 10 min). After blocking with 10% normal goat serum (10 min), the sections were incubated overnight in a humidified chamber at 4 °C with the primary antibodies. The dilutions of the previously generated und described primary antibodies are shown in supplementary table 1. Secondary antibodies (Cy3-conjugated goat-anti-rabbit IgG, catalogue code 111–165-144, dilution 1/1000 and FITC-conjugated goat-anti-mouse IgG, catalogue code 115–095-068, dilution 1/100, both from Jackson Immuno Research Laboratories, West Grove, PA) were applied for 2 h at room temperature and sections were finally mounted using DABKO-glycergel and coverslips. Images were acquired at a Leica DM6000 B fluorescence microscope on Leica DFC350 FX fluorescence monochrome digital camera (Leica Microsystems, 35578 Wetzlar, Germany).

### Western blot analysis

#### Sample preparation

Kidneys were homogenized in ice-cold lysis buffer (Mannitol 200 mM, HEPES 80 mM, KOH 41 mM) containing protease inhibitor (Complete Ultra, Roche) and phosphatase inhibitor (PhosSTOP, Roche) using MagNA Lyser Green Beads (Roche) and Precellys 24 tissue homogenizer (Bertin Instruments), 2 × 20 s (2000 *g*). The homogenized samples were centrifuged for 10 min (1700 *g*) and the protein-containing supernatant was removed and stored at − 80 °C.

#### SDS-PAGE and Western blot

Equal amounts of protein (25 μg) from kidney samples were denatured in Laemmli Buffer (SDS, β-mercaptoethanol, bromophenol blue, glycerol and 0.5 M Tris-HCl buffer, pH 6.8), separated by SDS-page, and electrophoretically transferred onto nitrocellulose membranes. The membranes were blocked with Odyssey Blocking solution (LI-COR Biosciences) (10 min) and then incubated at 4 °C for 16 h with the primary antibodies (see table 1 of supplementary data) diluted in Odyssey Blocking buffer: PBS (1:5). After repeated washes with PBS, blots were incubated at room temperature for 2 h with secondary antibodies (goat-anti-rabbit IRDye 800, product number 926-32211 and goat-anti-mouse IRDye 680, product number 926-32220, 1:20′000, both from LI-COR Biosciences), in Casein Blocking solution: H2O (1:10). Immunoreactive bands were visualized and quantified with the Odyssey IR imaging system and software (LI-COR Biosciences) and normalized for either β-actin or the total protein signal obtained by REVERT Total Protein Stain (LI-COR Biosciences).

### RNA isolation and quantitative PCR

#### RNA isolation and cDNA generation from total kidney tissue

Total RNA from kidney tissue was isolated using Promega kit (SV Total RNA Isolation System, catalog number Z3100), according to the manufacturer’s protocol. cDNA was generated using Promega kit (GoScript™ Reverse Transcription System, catalog number A5000) as per the manufacturer protocol. For whole kidney tissue, cDNA was further diluted to 1:5 in H_2_O.

#### RNA isolation and cDNA generation from isolated DCTs

Manually microdissected tubules were frozen in RNA lysis buffer containing β-mercaptoethanol, and RNA was isolated using Absolutely RNA Nanoprep kit (Agilent Technologies, catalog number 400753), following manufacturer’s protocol. cDNA was generated using Sensifast cDNA Synthesis kit (Bioline, catalog number BIO65053), and undiluted cDNA was used for RT-qPCR.

#### Real-time quantitative PCR

Expression levels of the genes of interest were quantified by real-time quantitative PCR using Light Cycler II 480 (Roche). Real-time PCR reactions were prepared using 5.0 μl of LightCycler 480 SYBR Green I Master (Roche), 3.75 μl of RNase free water, 0.25 μl of each primer (100 μM), and 0.75 μl of cDNA (final reaction volume of 10 μl). Gene-specific primer sequences are listed in table 2 of supplementary data. Specificity of the primers was first tested in a standard PCR with subsequent separation of PCR products in a 2% agarose gel. Relative mRNA gene expression was measured after normalization to GAPDH gene expression (total kidney) and ribosomal RNA gene expression (isolated DCTs).

### Manual microdissection of distal convoluted tubules

Manual microdissection of distal convoluted tubules was conducted according to a previously published protocol [47]. After perfusion of anesthetized mice with MEM media (GibcoBRL, Ref number 11058021) via left ventricle, the kidney was sagittally cut into two halves and the medulla was excised. Cortex was sliced into 1-mm thin sections that were then incubated in a digestion solution containing 4 ml MEM, 5 mM glycine, 6 mg/ml trypsin inhibitor (Trypsin inhibitor from Glycine max, Sigma-Aldrich, T9128), and 250 μg/ml collagenase (Collagenase from Clostridium histolyticum, Sigma-Aldrich, C9891), pH 7.4 at 37 °C for 25 min without shaking. The digested cortex sections were subsequently washed with ice-cold HEPES solution (NaCl 125 mM, KCl 3 mM, MgSO_4_ 1.2 mM, CaCl_2_ 1 mM, KH_2_PO_4_ 2 mM, glucose 5 mM, HEPES 32.2 mM), and the renal DCTs were manually microdissected under a stereomicroscope based on their distinct morphological characteristic.

### Implantation of mini-osmotic pump

#### Loading mini-osmotic pumps

Bumetanide (Sigma-Aldrich, B3023) (dosage of 40 mg/kg body weight/day) and bromodeoxyuridine (Sigma-Aldrich, B5002) (dosage 40 mg/kg body weight/day) were dissolved in H_2_O: PEG300 (1:5) with incubation on a thermo shaker on 36 °C, 1000 rpm, overnight. Mini-osmotic pumps from Alzet® (model 2001, DURECT Corporation, Cupertino, CA, USA) with a filling volume of 200 μl and a pump delivery rate of 1 μl/h were used. One day prior to pump implantation, the mini-osmotic pumps were loaded with appropriate volume (200 μl) of the solution as directed by manufacturer using the provided needle. The loaded mini-osmotic pumps were primed in sterile NaCl 0.9% at 37 °C overnight according to the supplier’s instruction.

#### Surgical implantation in mice

Mice were anesthetized with isoflurane and buprenorphine 0.01 mg/kg body weight (Temgesic®, 0.3 mg/ml, Indivior Schweiz AG, 3015880). For implantation of the pump, mice were placed in a prone position. A skin incision (1–2 cm) was made across the neck and a subcutaneous pocket was created using blunt forceps. After inserting the pump into the subcutaneous pouch, the skin wound was closed with two surgical clips.

### Semi-thin sections

For semi-thin sectioning, kidneys were fixed by vascular perfusion with 3% PFA as described above and postfixed in the same fixative to which 1% glutaraldehyde was added. The tissue was embedded in epoxy resin (EPON) and semi-thin sections of 0.8 μm were cut with an ultramicrotome (Leica UltraCut E ultramicrotome). For staining, slides were incubated at 60 °C for 2 min in 0.5% toluidine blue (Merck, 11593) and 0.5% sodium tetraborate decahydrate (Sigma-Aldrich, S9640), diluted in water, followed by coverslip mounting with Entellan new (Merck 1079610100).

### Morphometric measurements

The fractional cortical tubular volume of DCT segments was assessed according to a previously described protocol [38]. Paraffin sections of kidneys were immunostained with an antibody against NCC (table 1 of supplementary material) and images were taken from each kidney section with the × 10 objective of the fluorescence microscope. DCTs were identified according to the antibody-staining pattern and the fractional cortical tubular volumes for DCTs were determined using a planimetric point-counting method performed with a computerized image analysis system (Stereo Investigator, MBF Bioscience, Williston USA).

### Statistical analysis

Unpaired two-tailed *t* test and 2-way ANOVA were used to compare the groups (GraphPad Prism, Version 8.0.2). Data are given as means ± SEM. Values were considered as significantly different when *p* < 0.05.

## Results

### Development and characterization of a new mouse model for inducible DCT-specific gene targeting

For a DCT-specific deletion of Prox-1, we first developed and characterized a novel mouse model with a tamoxifen-inducible cre activity in the DCT (see “Material and methods”). Upon tamoxifen administration, these mice show a strong nuclear localization of the cre recombinase specifically in the DCT (Fig. 1B), comprising both DCT1 and DCT2 as identified by their characteristic staining pattern for NCC and calbindin D28K [24]. Using a tdTomato-red reporter mouse line [26], we found that cre-mediated recombination is negligible without tamoxifen induction, but increases drastically in cortical tubules when tamoxifen is administered for 5 days. Higher magnifications showed that the cre-mediated recombination starts precisely at the transition from TAL to DCT and ends at the transition from DCT to CNT (Fig. 1C). Immunoblotting confirmed that the insertion of the cre-ERT2 in the 3′UTR of NCC does not interfere with NCC expression and phosphorylation (Fig. 1D, E). Thus, this new NCC-cre-ERT2 mouse model allows a timed gene targeting restricted to the DCT. Unlike to a recently published mouse model with constitutive cre expression under the control of the NCC promoter [44], the new mouse model does not show evidence for cre-mediated recombination in non-DCT cells. Similar observations were made by the group of Jim McCormick who just reported a similar NCC-cre-ERT2 mouse model generated by a similar gene-modification strategy as we employed [5].

### Prox-1 is enriched in adult mouse DCT

**Fig. 2:**
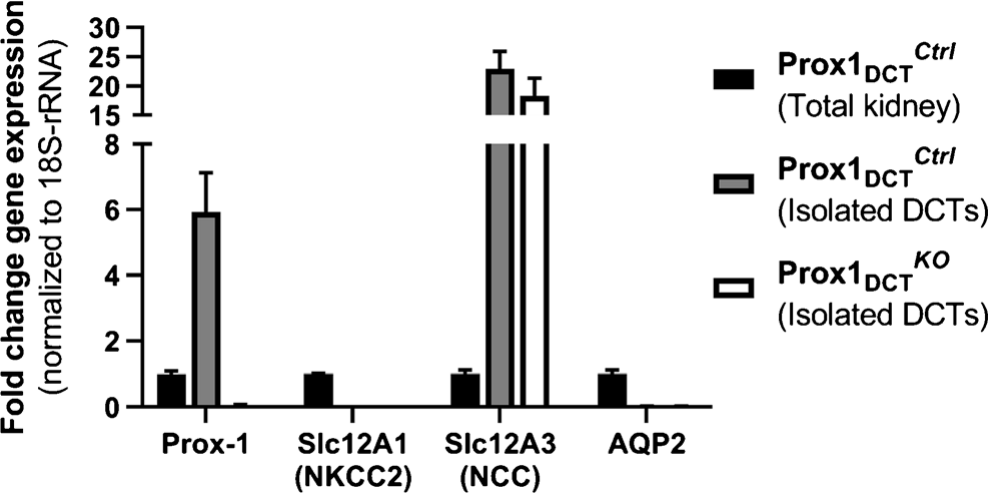
Prox-1, Slc12A1/NKCC2, Slc12A3/NCC, and AQP2 mRNA expression in Prox-1_DCT_^*Ctrl*^ versus Prox-1_DCT_^*KO*^ isolated DCTs and in total kidney tissue. Prox-1: Prox-1 gene expression is significantly increased in isolated DCTs (Prox-1_DCT_^Ctrl^) compared to whole kidney tissue (Prox-1_DCT_^Ctrl^) (*p* = 0.0035). Confirmation of a Prox-1 deletion with almost none Prox-1 mRNA expression in isolated DCTs from Prox-1_DCT_^*KO*^ mice compared to isolated DCTs from Prox-1_DCT_^Ctrl^ mice (*p* = 0.000006). Slc12A1/NKCC2, AQP2: No contamination of isolated DCTs (Prox-1_DCT_^*Ctrl*^ and Prox-1_DCT_^*KO*^) with other nephron segments, confirmed by absent Slc12A1/NKCC2 and AQP2 gene expression in DCT samples. Slc12A3/NCC: 20–30-fold higher Slc12A3/NCC expression in isolated DCTs compared to total kidney tissue. Kidneys and renal tubules were isolated 1 week after start of tamoxifen induction. Data are mean ± SEM, expressed as fold difference compared to the gene expression in the total kidney group, *n* = 5

Prox-1 mRNA expression was determined in manually dissected DCTs from both Prox-1_DCT_^Ctrl^ and Prox-1_DCT_^KO^ mice as well as from total kidney homogenates from Prox-1_DCT_^Ctrl^ mice. Comparing Prox-1 mRNA abundance in isolated DCT segments (ctrl) and total kidney tissue (ctrl), Prox-1 mRNA transcript levels showed a 5–6-fold enrichment in the DCT (Fig. 2). In the Prox-1_DCT_^KO^ mice, hardly any Prox-1 expression was detectable in the isolated DCTs, confirming the efficacy of the DCT-specific Prox-1 deletion in our mouse model (Fig. 2). Isolated DCTs were not contaminated with other tubular segments, confirmed by absent Slc12A1/NKCC2 and AQP2 gene expression.

### No gross pathological characteristics in Prox-1_DCT_^KO^ mice

Breeding of NCC-cre-ERT2 with floxed Prox-1 animals resulted in a normal Mendelian inheritance pattern. Cre-induction and excision of the Prox-1 gene in DCT cells were induced upon tamoxifen administration either immediately after birth or at the age of weaning. Independent from the time point of induction, all mice with DCT-specific Prox-1 deletion (Prox-1_DCT_^KO^) did not show a gross pathological phenotype. Body weight, food, and water intake and 24-h urine excretion were comparable among the genotypes (Table 1).

**Table 1:** Metabolic parameters, plasma and urine electrolyte concentrations and plasma aldosterone, 11-deoxycorticosterone, and corticosterone in Prox-1_DCT_^KO^ mice

	Prox1_DCT_^Ctrl^	Prox1_DCT_^*KO*^
Age (days)	90 ± 1	100 ± 3
Body weight (g)	31.3 ± 0.9	31.6 ± 1.0
Food intake (g/day)	7.3 ± 0.6	5.9 ± 0.3
Water intake (ml/day)	2.6 ± 0.6	3.3 ± 0.8
Urine volume (ml/day)	1.2 ± 0.3	1.1 ± 0.3
Plasma Na^+^ (mmol/l)	149.4 ± 0.6	150.9 ± 1.3
Plasma K^+^ (mmol/l)	4.0 ± 0.1	4.1 ± 0.2
Plasma Ca^2 + −^ (mmol/l)	1.26 ± 0.03	1.25 ± 0.03
Plasma Mg^2+^ (mmol/l)	0.85 ± 0.03*	0.76 ± 0.03*
Plasma HCO3^−^ (mmol/l)	19.3 ± 0.5	20.0 ± 0.5
Plasma PO4^3−^(mmol/l)	1.61 ± 0.12	1.83 ± 0.15
Plasma aldosterone (nM)	0.32 ± 0.04	0.37 ± 0.09
Plasma 11-Deoxycorticosterone (nM)	1.38 ± 0.36	0.98 ± 0.37
Plasma corticosterone (nM)	107.7 ± 20.8	92.9 ± 19.0
Urine Na^+^/urine creat. (mmol/mmol)	40.8 ± 6.0	41.4 ± 4.0
Urine K^+^/urine creat. (mmol/mmol)	179.4 ± 17.2	147.6 ± 11.3
Urine Ca^2+^/urine creat. (mmol/mmol)	0.84 ± 0.14	0.60 ± 0.12
Urine Mg^2+^/urine creat. (mmol/mmol)	3.70 ± 1.13	1.83 ± 0.77

Prox-1_DCT_^*KO*^ mice exhibit a significant hypomagnesemia (*p* = 0.036) compared to the Prox-1_DCT_^*Ctrl*^ group. For other metabolic parameters, plasma electrolytes, plasma aldosterone, 11-deoxycorticosterone, corticosterone, and urinary ion excretion, no difference was detected between the genotypes. Values are means ± SEM. *n* = 11 (plasma electrolytes), *n* = 13 (plasma Mg^2+^), *n* = 7 (aldosterone, 11-deoxycorticosterone, and corticosterone), *n* = 5 (body weight, food and water intake, urine excretion, urine electrolytes)

### No effect of a Prox-1 deletion on DCT development and outgrowth

**Fig. 3:**
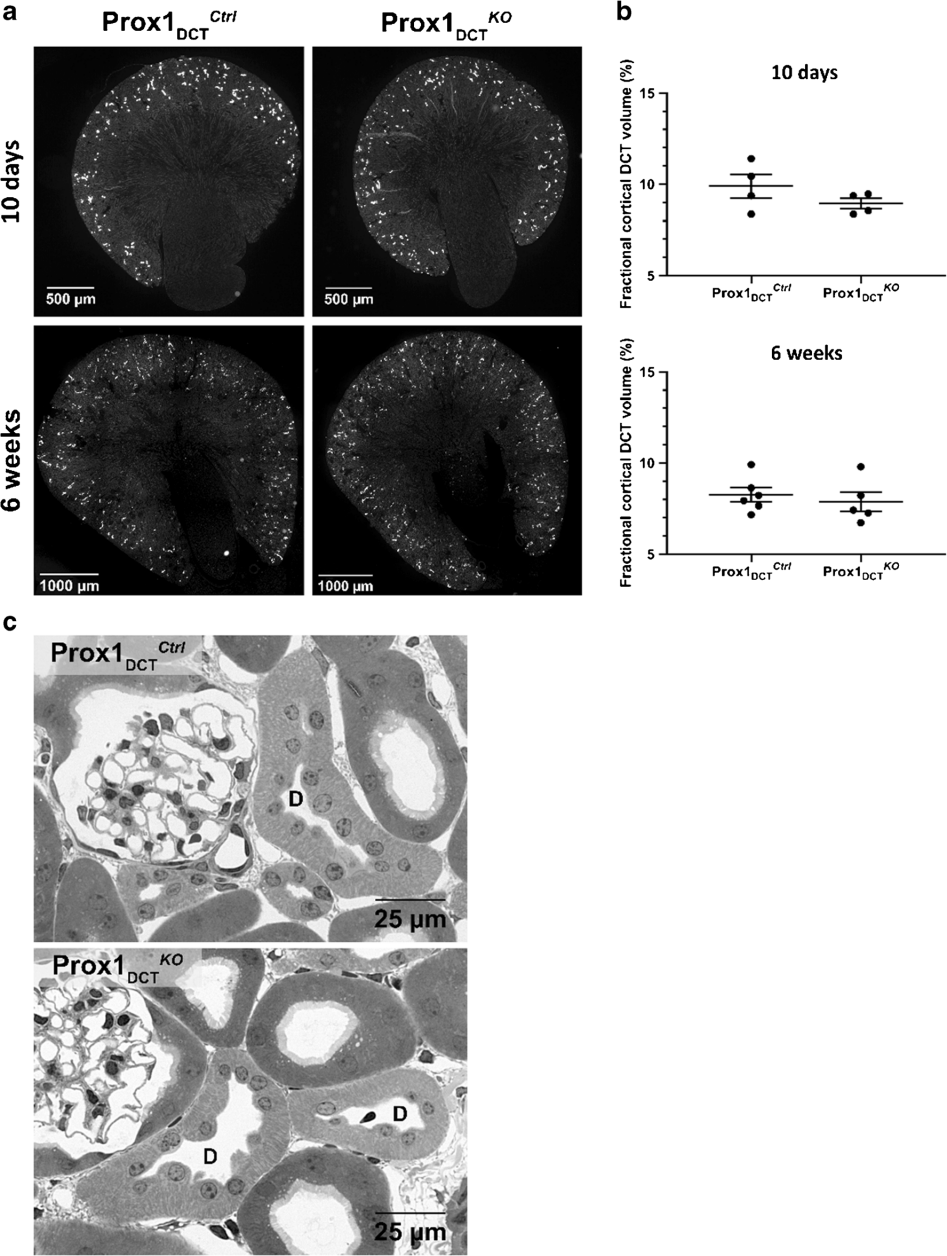
DCT morphology and fractional cortical volume of DCT in Prox-1_DCT_^Ctrl^ versus Prox-1_DCT_^KO^ mice at age 10 days and 6 weeks. **A** NCC protein expression (immunostaining on paraffin sections) in kidneys of 10-day-old pups (upper panel) and 6-week-old (lower panel) Prox-1_DCT_^Ctrl^ versus Prox-1_DCT_^KO^ mice. Cre recombination and deletion of Prox-1 in the DCT was induced immediately after birth. **B** No difference in the fractional cortical volume (%) of whole DCT in Prox-1_DCT_^Ctrl^ versus Prox-1_DCT_^KO^ mice was observed upon deletion of Prox-1, neither in pups nor in 6-week-old mice. DCTs were identified due to NCC immunostaining. Values are means ± SEM. *n* = 4 (10 days, ctrl + ko), *n* = 6 (6 weeks, ctrl), *n* = 5 (6 weeks, ko). **C** Distal convoluted tubules (D) in the renal cortex of Prox-1_DCT_^Ctrl^ and Prox-1_DCT_^KO^ mice reveal a normal DCT morphology (adult kidneys) in both genotypes, epon semi-thin sections

To address the question whether Prox-1 influences the outgrowth of the DCT during renal development, Prox-1 deletion was induced in newborn mice via tamoxifen gavage of the breastfeeding mother during the first 5 days after birth. So-treated mice were sacrificed 10 days and 6 weeks after birth and kidneys were collected for further analysis. Based on paraffin sections of kidneys immunostained with antibody against NCC, DCTs were identified and the fractional cortical tubular volume for DCTs was determined using a planimetric point-counting method (morphometry). Neither a change in kidney morphology nor in the cortical tubular DCT volume was observed in Prox-1_DCT_^KO^ mice compared to Prox-1_DCT_^Ctrl^ mice both 10 days and 6 weeks after birth (Fig. 3A, B). Morphologic analysis of the renal cortex (epon semi-thin sections) in both control and Prox-1_DCT_^KO^ mice revealed no structural abnormalities of the DCT epithelium in response to a genetic loss of Prox-1 (Fig. 3C).

### Deletion of Prox-1 does not influence DCT cell proliferation rate in response to a 3-day treatment with bumetanide

**Fig. 4:**
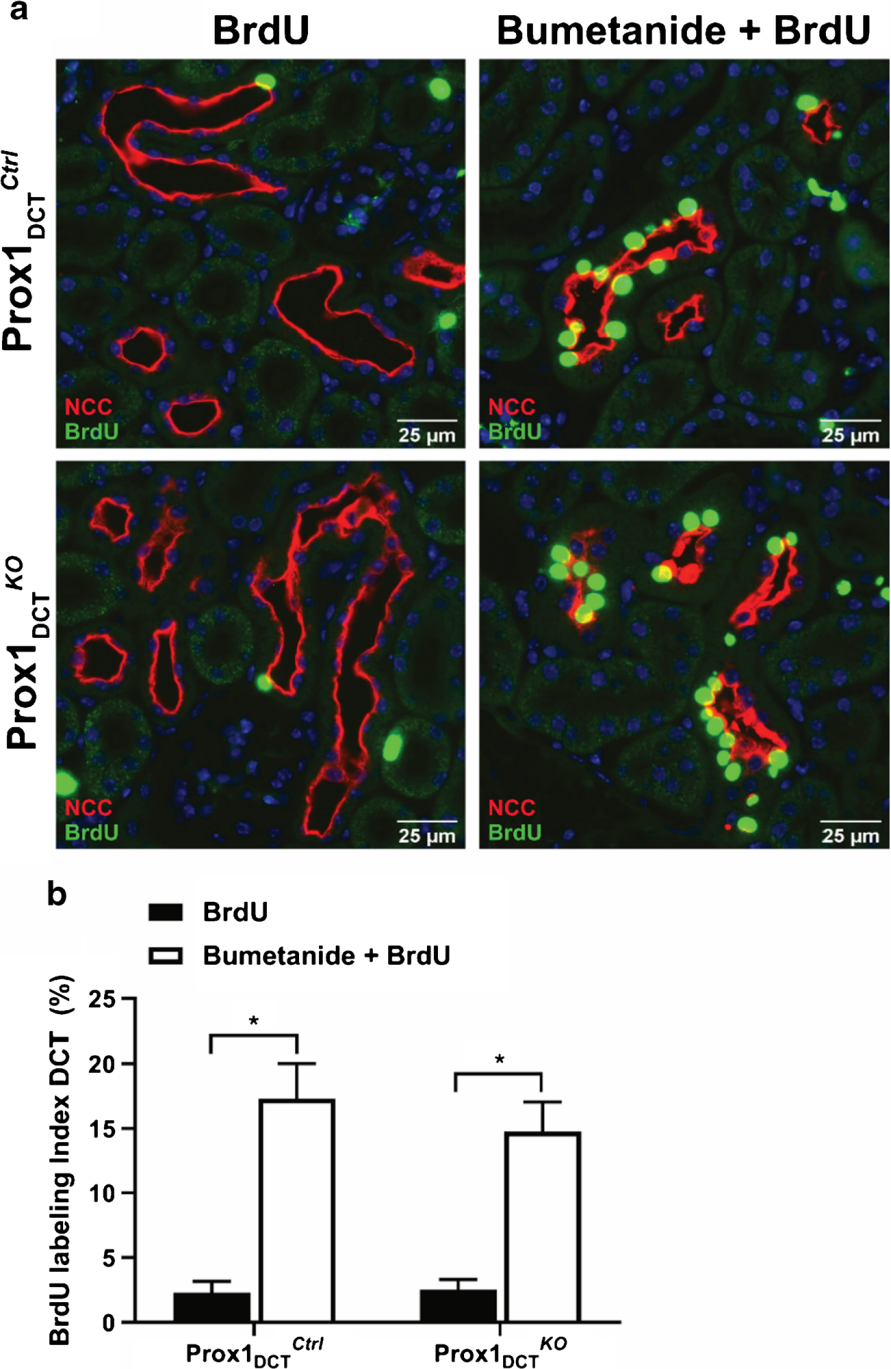
DCT cell proliferation rate in Prox-1_DCT_^Ctrl^ versus Prox-1_DCT_^KO^ mice in response to a 3-day treatment with bumetanide. **A** Paraffin sections of Prox-1_DCT_^Ctrl^ mice kidneys (upper panel) and Prox-1_DCT_^KO^ mice kidneys (lower panel) after a 3-day treatment with bumetanide and BrdU (right) or BrdU only (left). A double-immunostaining for NCC (red) and BrdU (green) allowed for the identification of DCT segments and proliferating DCT cells. **B** A significant increase in DCT cell proliferation rate was achieved in Prox-1_DCT_^Ctrl^ (*p* = 0.0001) and Prox-1_DCT_^KO^ (*p* = 0.0007) by the administration of bumetanide and BrdU compared to the situation where mice received BrdU only. However, no difference in the proliferation rate between the different genotypes was observed. Data are mean ± SEM, *n* = 6 (BrdU only), *n* = 5 (Bumetanide + BrdU, ctrl), *n* = 4 (Bumetanide + BrdU, ko)

To test whether Prox-1 is involved in epithelial remodeling processes of the DCT that occur in response to a loop diuretic treatment, bumetanide was applied continuously during 3 days. The simultaneous administration of bromodeoxyuridine (BrdU) allowed for the detection of DCT cell proliferation rate. After the bumetanide treatment, a significant increase in DCT cell proliferation rate was observed in the group that received bumetanide plus BrdU compared to the group that was treated with BrdU only (Fig. 4A). However, quantitative analysis of the percentage of proliferating DCT cells under the bumetanide treatment revealed no difference in the proliferation rate between Prox-1_DCT_^KO^ versus Prox-1_DCT_^Ctrl^ mice (Fig. 4B).

### Overt hypomagnesemia, but no other changes in plasma electrolytes and urinary ion excretion in Prox-1_DCT_^KO^ mice

Analysis of plasma electrolytes revealed a significant hypomagnesemia in Prox-1_DCT_^KO^ mice, whereas no differences in other plasma electrolyte concentrations (Na^+^, K^+^, Ca^2+^, phosphate) and plasma aldosterone, 11-dehydrocorticosterone and corticosterone levels between the knockout and control groups were detected (Table 1). Urinary ion excretion (Na^+^, K^+^, Ca^2+^, Mg^2+^) was similar in Prox-1_DCT_^Ctrl^ and Prox-1_DCT_^KO^ mice (Table 1).

### Decreased NCC abundance due to a loss of Prox-1 in the DCT

**Fig. 5:**
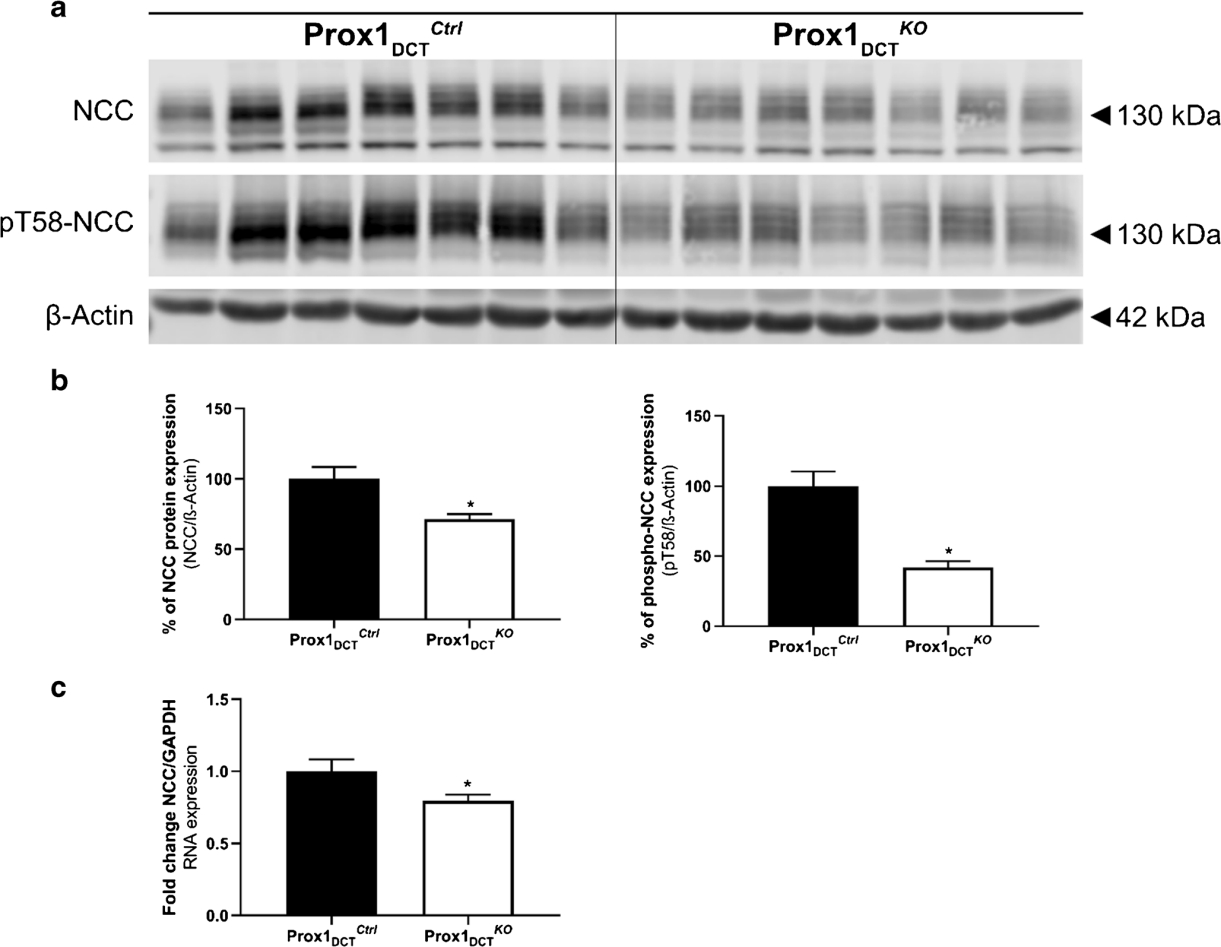
NCC and phospho-NCC protein and mRNA abundance in Prox-1_DCT_^Ctrl^ versus Prox-1_DCT_^KO^ mice. **A** Protein expression of total NCC and pT58-NCC in total kidney homogenates of Prox-1_DCT_^Ctrl^ versus Prox-1_DCT_^KO^ mice. **B** Densitometry analysis reveals a significant downregulation of total NCC (*p* = 0.0083) and in pT58-NCC (*p* = 0.0002) protein expression in Prox-1_DCT_^KO^ compared to the control group. Data are mean ± SEM, *n* = 7 **C** Gene expression of NCC in total kidney homogenates of Prox-1_DCT_^Ctrl^ versus Prox-1_DCT_^KO^ mice reveals a significant downregulation of mRNA (*p* = 0.049) in Prox-1_DCT_^KO^ compared to the control group. Data are mean ± SEM, *n* = 7. Kidneys were studied 6 weeks after start of tamoxifen induction

Deletion of Prox-1 in the DCT in adult mice for 6 weeks resulted in a significant downregulation of total NCC and phospho-NCC protein abundance in total kidney homogenates of Prox-1_DCT_^KO^ compared to Prox-1_DCT_^Ctrl^ (Fig. 5A, B). Similar results were shown for NCC gene expression (Fig. 5C). Interestingly, downregulation of NCC mRNA was less pronounced at 1 week after tamoxifen induction (Fig. 2), suggesting that the downregulation of NCC at the mRNA level is not an immediate effect.

### No change in ENaC protein expression in Prox-1_DCT_^KO^ mice

**Fig. 6:**
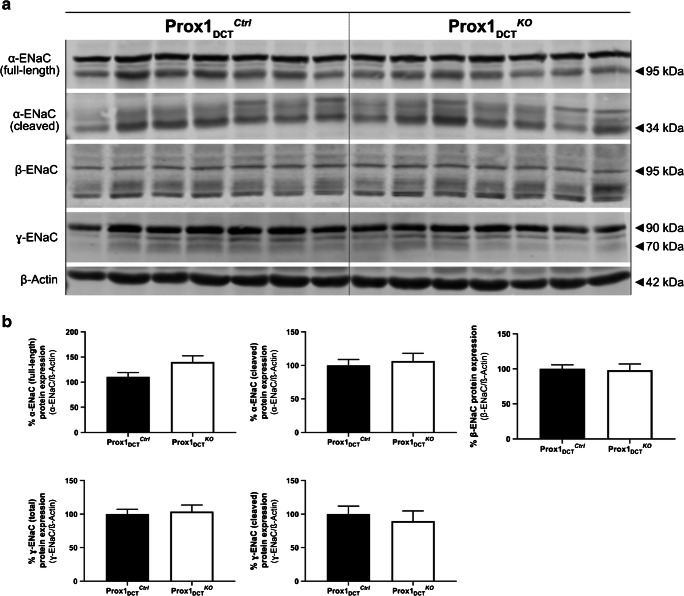
ENaC protein abundance in Prox-1_DCT_^Ctrl^ versus Prox-1_DCT_^KO^ mice. **A** Protein expression of α-, β-, and ɣ-ENaC in total kidney homogenates of Prox-1_DCT_^Ctrl^ versus Prox-1_DCT_^KO^ mice. **B** Densitometry analysis reveals no significant regulation of ENaC in Prox-1_DCT_^KO^ compared to the control group. Data are mean ± SEM, *n* = 7. Kidneys were studied 6 weeks after start of tamoxifen induction

Protein expression analysis for α-, β-, and ɣ-ENaC revealed no significant difference in kidneys of Prox-1_DCT_^Ctrl^ and Prox-1_DCT_^KO^ mice for both the total/glycosylated and the cleaved forms of ENaC (Fig. 6A, B).

### Reduced protein expression of the DCT-specific Mg^2+^ channel TRPM6 in Prox-1_DCT_^KO^ mice

**Fig. 7:**
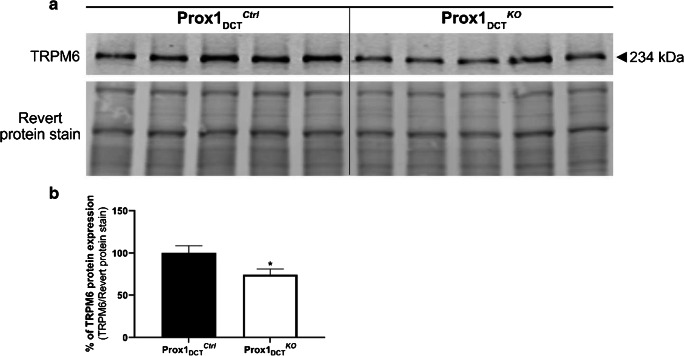
TRPM6 protein and mRNA abundance in Prox-1_DCT_^Ctrl^ versus Prox-1_DCT_^KO^ mice. **A** Protein expression of TRPM6 in total kidney homogenates of Prox-1_DCT_^Ctrl^ versus Prox-1_DCT_^KO^ mice. **B** Densitometry analysis reveals a significant down-regulation of TRPM6 (*p* = 0.0317) protein expression in Prox-1_DCT_^KO^ compared to the control group. Data are mean ± SEM, *n* = 5. Kidneys were studied six weeks after start of tamoxifen induction

**Fig. 8:**
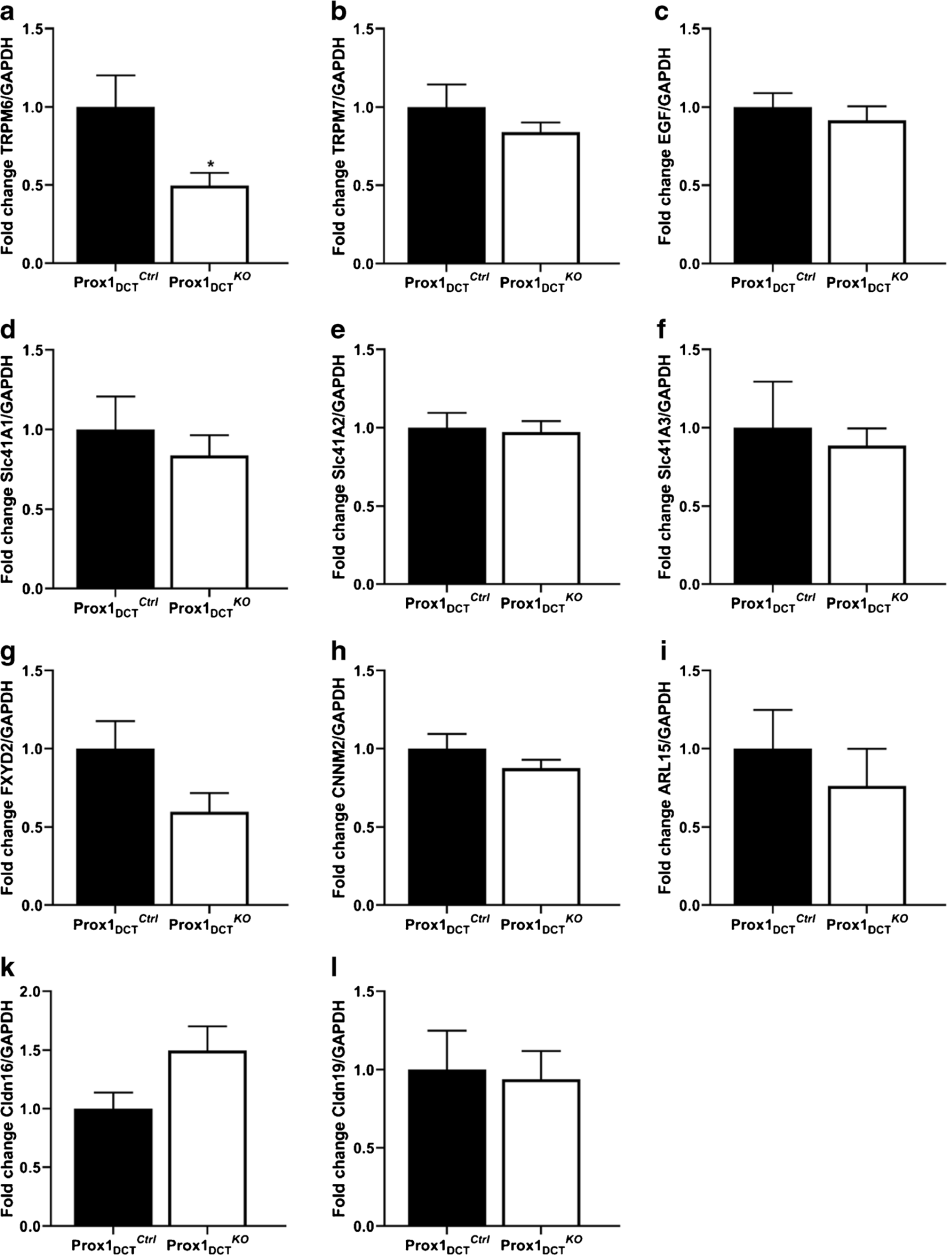
mRNA expression of Mg^**2**+^ channels and regulatory proteins involved in Mg^**2**+^ homeostasis in Prox-1_DCT_^Ctrl^ versus Prox-1_DCT_^*KO*^ mice (total kidney homogenates). **A** TRPM6 mRNA expression is significantly downregulated in Prox-1_DCT_^KO^ mice (*p* = 0.039). mRNA expression of **B** TRPM7, **C** EGF, **D** Slc41A1, **E** Slc41A2, **F** Slc41A3, **G** FXYD2, **H** CNNM2, **I** ARL15, **K** Cldn16, **L** Cldn19 in total kidney homogenates is similar in Prox-1_DCT_^Ctrl^ versus Prox-1_DCT_^KO^ mice. Relative gene expression was analyzed with normalization against GAPDH expression (housekeeping gene). Data are mean ± SEM, expressed as fold difference compared to the gene expression in the control group, *n* = 7. Kidneys were studied 6 weeks after start of tamoxifen induction

A significant reduction in the protein expression of the DCT-specific Mg^2+^ channel TRPM6 was observed in total kidney homogenates of Prox-1_DCT_^KO^ mice (Fig. 7A, B). In parallel, TRPM6 mRNA expression was also significantly reduced (Fig. 8A). mRNA levels of other renal magnesium transporters and proteins involved in Mg^2+^ homeostasis (EGF, CNNM2, EGF, FXYD2, Cldn16, Cldn19, TRPM7, Slc41a1–3) in total kidney homogenates were not altered in Prox-1_DCT_^KO^ mice compared to their control littermates (Fig. 8B–L).

## Discussion

Transcription factors are proteins that bind to particular DNA sequences in gene regulatory regions and control gene expression and activity [22]. The human genome encodes about 1600 regulatory sequence-specific transcription factors that have been identified and functionally characterized [15]. With the exception of the steroid receptors (e.g., the mineralocorticoid receptor), most studies addressing the role of transcription factors in the kidney focus on their significance during embryonic development and their regulatory function in nephrogenesis and differentiation of nephron progenitor cells [3]. Accordingly, gene mutations in transcription factors have been associated with congenital renal malformations and inherited diseases, emphasizing their important role during renal embryogenesis. For example, mutations in the transcription factor hepatocyte nuclear factor 1 homeobox B (HNF1B) gene have been shown to cause renal malformations, hypomagnesemia, and maturity-onset diabetes of the young (MODY) [1, 20]. Part of this phenotype was suggested to be related to a transcriptional downregulation of the basolateral Kir4.1/Kir5.1 K^+^ channel in the DCT [19]. In contrary to the evolving knowledge of the role of transcription factors during renal development, only little is known about transcriptional control and regulation of gene expression in the adult kidney. Interestingly, the DCT transcriptome established previously [7, 23, 33] revealed a high number of enriched DCT genes known to be involved in transcriptional regulation and growth control during renal development and/or tumorigenesis, suggesting that this “embryological” transcriptome contributes to the characteristic adaptation capacity and plasticity exhibited by the DCT in the adult stage.

Given its regulatory function in cell proliferation in other organs such as the retina [8], muscle tissue [18], central nervous system [16], and lymphatic tissue [49], we hypothesized that Prox-1 might play a role in DCT epithelium structural remodeling processes that occur in response to a loop diuretic treatment. However, after a 3-day treatment with bumetanide, no difference in the DCT cell proliferation rate was observed in the Prox-1_DCT_^KO^ mice compared to the Prox-1_DCT_^Ctrl^ group. A direct effect of Prox-1 on DCT cell proliferation was therefore not verifiable. Consistently, fractional cortical DCT volume remained unchanged subsequent to a loss of Prox-1, also indicating that Prox-1 is not directly involved in structural epithelial remodeling processes of the DCT.

In the developing mouse kidney, Prox-1 was demonstrated to be highly expressed in distal renal tubules, contributing to the differentiation of the medullary part of the loop of Henle [17]. Given that a main portion of the outgrowth of the NCC-positive rodent DCT takes place in the first 10 days perinatally [37, 38], we investigated the effect of a Prox-1 deletion in the DCT at this early time of life. Mice in which a Prox-1 deletion was introduced during the first 10 days after birth did not reveal any morphological renal abnormality and the cortical tubular DCT volume remained intact, indicating that a loss of Prox-1 in this period of nephron development does not affect DCT outgrowth. Hence, Prox-1 may be less important for the initial formation of the DCT, but rather contributes to the regulation of DCT function and ion transport at the full-grown stage.

The expression of NCC as the major sodium reabsorption pathway of the DCT was significantly decreased due to a loss of Prox-1, confirming that Prox-1 does play a modulatory role in DCT function and ion transport regulation. As no change in the cortical tubular DCT volume was observed in Prox-1_DCT_^KO^ mice neither during renal development nor at adulthood, we assume that this NCC downregulation is not due to a loss of DCT epithelium surface, but due to a NCC downregulation on the cellular level, which occurs as a consequence of the genetic disruption of Prox-1. The underlying gene networks modified by Prox-1 and resulting in this NCC downregulation remain elusive. To date, the regulation of NCC expression and phosphorylation has been understood as a complex interplay of kinases, phosphatases and ubiquitin-ligases [13, 34, 41, 51]. Here, we support the concept that—in addition to the already known posttranslational regulatory mechanisms—transcription factors previously linked to developmental processes but not epithelial transport, may participate in the control of DCT function.

Although the abundance of NCC as the major sodium reabsorption pathway of the DCT is significantly reduced in Prox-1_DCT_^KO^ mice, no functional consequences in renal sodium handling, especially no salt-wasting phenotype was observed. Consistently, NCC null mutations lead only to mild perturbation of sodium and fluid volume homeostasis in mice [39] and most human patients [40]. As almost every nephron segment participates in renal sodium reabsorption, compensatory mechanisms in other tubular segments that counteract NCC downregulation in the DCT are very likely to occur, even though no change in ENaC protein expression was demonstrated in our study.

The DCT is also a key site for Mg^2+^ balance and reabsorbs 10% of filtered tubular Mg^2+^ in an active transcellular manner via the luminal Mg^2+^ channel TRPM6 [6, 28, 46]. With this, the DCT represents the predominant site of specifically regulated Mg^2+^ excretion and ultimately controls the amount of Mg^2+^ excreted in the urine, because no Mg^2+^ reabsorption occurs in downstream nephron segments [45]. Altered DCT structure and function either due to a genetic loss of NCC in Gitelman syndrome or an inhibition of NCC activity by thiazide-like diuretics may relevantly influence Mg^2+^ homeostasis and cause renal Mg^2+^ wasting and hypomagnesemia in mice [25, 31] and human patients [9, 21, 35]. Others and we previously reported a distinct reduction of TRPM6 abundance in a NCC null mutant mouse model, most probably linked to a loss of a significant portion of the DCT epithelium [31, 38]. In the current study, no change in the tubular DCT volume in Prox-1_DCT_^KO^ mice is observed. The reduced TRPM6 expression and hypomagnesemia can hence not be explained with a loss of TRPM6 expressing DCT epithelium. One can only speculate whether the hypomagnesemic phenotype and TRPM6 downregulation in the Prox-1_DCT_^KO^ mouse model is due to a direct or indirect regulation of TRPM6 through Prox-1 or whether this occurs secondary due to reduced NCC expression and diminished transcellular sodium transport.

Surprisingly, urinary magnesium excretion was unaltered or even slightly reduced in Prox-1_DCT_^KO^ mice despite an overt hypomagnesemia compared to the control group. This is consistent with findings in kidney-specific TRPM6 null mice, which show hypomagnesemia despite reduced urinary Mg^2+^ excretion [4]. It is conceivable that the downregulation of TRPM6-dependent Mg^2+^ transport in DCT gets compensated by an upregulation of paracellular Mg^2+^ reabsorption in the TAL and PT [28].

In conclusion, we developed a new mouse model for an inducible gene targeting in the renal DCT. We used this mouse model to study the DCT-specific role of the transcription factor Prox-1. While previous studies on Prox-1 and other renal transcription factors focused mainly on their role during embryonic development, the present study shows that Prox-1 has an important role in the adult DCT by controlling TRPM6-dependent Mg^2+^ homeostasis. The study may set an important base for future studies addressing the functional involvement of this and possibly other DCT-enriched transcription factors in regulatory pathways governing kidney function.

## Supplementary Information

ESM 1 (DOCX 20 kb)
