# Supplementary material for: Deletion of the transcription factor Prox-1 specifically in the renal distal convoluted tubule causes hypomagnesemia via reduced expression of TRPM6 and NCC
Source: Pflugers Arch. 2020 Nov 16;473(1):79–93. doi: 10.1007/s00424-020-02491-1 (PMC7782375; doi:10.1007/s00424-020-02491-1)
Supplement: Supplementary file 1 — (DOCX 20 kb) [file 424_2020_2491_MOESM1_ESM.docx]

**Supplementary Tables**

Supplementary Table 1: Antibodies used for immunohistochemical and western blot analysis.

| **Antibody** | **Host** | **Source** | **Reference** | **Dilution IF** | **Dilution WB** |
| --- | --- | --- | --- | --- | --- |
| Anti-NCC | rabbit | J. Loffing | [Sorensen et al, Kidney Int 2013] | 1/3000 | 1/500 1/5000 |
| Anti-pT53-NCC | rabbit | J. Loffing | [Sorensen et al, Kidney Int 2013] | - | 1 1/5000 |
| Anti-pT58-NCC | rabbit | J. Loffing | [Sorensen et al, Kidney Int 2013] | - | 1/5000 |
| Anti-α-ENaC | rabbit | J. Loffing | [Sorensen et al, Kidney Int 2013] | - | 1/5000 |
| Anti-β-ENaC | rabbit | J. Loffing | [Wagner et al, Am J Physiol Renal Physiol 2008] | - | 1/20’000 |
| Anti-​[ɣ](https://de.wikipedia.org/wiki/Liste_der_IPA-Zeichen)-ENaC | rabbit | J. Loffing | [Wagner et al, Am J Physiol Renal Physiol 2008] | - | 1/40’000 |
| Anti-TRPM6 | rabbit | J. Loffing | [Schnoz et al, Nephrol Dial Transplant 2019] | 1/3000 | 1/5000 |
| Anti-Cre recombinase | rabbit | G. Schütz | [Kellendonk C et al, J Mol Biol 1999] | 1/10’000 | - |
| Anti-calbindin D28k | mouse | Swant, Marly, CH | [Catalogue code 300] | 1/5000 | - |
| Anti-BrdU | mouse | BD Biosciences,  San Jose, CA | [Catalogue number 347580] | 1/50 | - |
| Anti-Actin | mouse | Sigma Aldrich  Saint Louis, MO | [Catalogue number A5316] | - | 1/5000 |

*References*

1. Sorensen MV, Grossmann S, Roesinger M, Gresko N, Todkar AP, Barmettler G, Ziegler U, Odermatt A, Loffing-Cueni D, Loffing J. (2013) Rapid dephosphorylation of the renal sodium chloride cotransporter in response to oral potassium intake in mice. Kidney Int. 83: 811-24.
2. Wagner CA, Loffing-Cueni D, Yan Q, Schulz N, Fakitsas P, Carrel M, Wang T, Verrey F, Geibel JP, Giebisch G, Hebert SC, Loffing J. (2008) Mouse model of type II Bartter's syndrome. II. Altered expression of renal sodium- and water-transporting proteins. Am J Physiol Renal Physiol. 294: F1373-80.
3. Schnoz C, Carrel M, Loffing J. (2020) Loss of sodium chloride co-transporter impairs the outgrowth of the renal distal convoluted tubule during renal development. Nephrol Dial Transplant. 35: 411-432.
4. Kellendonk C, Tronche F, Casanova E, Anlag K, Opherk C, Schütz G. (1999) Inducible site-specific recombination in the brain. J Mol Biol. 285: 175-82.

Table 2: Primers used for qPCR analysis.

| **Gene** | **Forward primer (5’-3’)** | **Reverse primer (5’-3’)** |
| --- | --- | --- |
| Prox-1 | GTTCACCAGCACACCCACCCAG | GATAGCCCTTCCTGCATTGCGCT |
| TRPM6 | ACTCAGAGCAGTTTGGCCAGCT | CGCAGACCTCCAGAGACCGC |
| TRPM7 | GGACCTTATGTAATGATGATTGGAA | AAGCAATACAAGAGCCATTATCAC |
| Slc12A3/NCC | TGACCTGCATTCATTCCTCA | GAAGCGAACAGGTTCTCCAG |
| Slc12A1/NKCC2 | GGAATTGGTCTGGGCGTCA | ATTGACCCACCGAACTCAGG |
| Slc9A3/NHE3 | TCTGTTTGTCAGCACCACTCTCA | TCACGATGCTCGCTCCTCTTCA |
| AQP2 | CCGCCATCCTCCATGAGATT | TGCATTGTTGTGGAGAGCAT |
| Scnn1a/α-ENaC | ACCCCGTGAGTCTCAACATC | CCTGGCGAGTGTAGGAAGAG |
| Scnn1b/β-ENaC | TTCAACTGGGGCATGACAG | CCGATGTCCAGGATCAACTT |
| Scnn1g/[ɣ](https://de.wikipedia.org/wiki/Liste_der_IPA-Zeichen)-ENaC | AGTTCAGAAAGAACTCTGCAGGC | GTGTTCAGGCAGTACCAATGC |
| EGF | GGGATGTGGGGGACTTACTAC | TGGCTCATCACAAGGGTT |
| Slc41A1 | GAGGGGGAGGAGTGATGAGA | TGTGGAACACCTGCGCCTTG |
| Slc41A2 | TGGCATGGTTTTGGACATAG | AGCGTCATTTCCAAGTTTCC |
| Slc41A3 | TGAAGGGAAACCTGGAAATG | GGTTGCTGCTGATGATTTTG |
| FXYD2 | CCGATGGCTGGGGAAATATCA | TCATAGTCGTACTCGAAGGGA |
| CNNM2 | GTCTCGCACCTTTGTTGTCA | GTCGCTCCGACTGAGAGAAT |
| ARL15 | TCCAGAATGCCGTTTTGAAT | AGTAGCGGCTCCAGTATTTCC |
| Cldn16 | GTTGCAGGGACCACATTAC | GAGGAGCGTTCGACGTAAAC |
| Cldn19 | GGTTCCTTTCTCTGCTGCAC | CATCCCACACGCCTTCCTGC |
| GAPDH | CCATCACCATCTTCCAGGAG | TCCATGGTGGTGAAGACAC |
| 18S-rRNA | GCAATTATTCCCCATGAACG | GGGACTTAATCAACGCAAGC |
